# Health hazards associated with LNG tank truck transportation accidents in China: a text-mining analysis of accident reports and risk-management implications

**DOI:** 10.3389/fpubh.2026.1952556

**Published:** 2026-09-10

**Authors:** Na Li, Yidan Tang, Jianghong Zhao

**Affiliations:** 1 School of Economics and Management, Southwest Petroleum University, Chengdu, Sichuan, China; 2 School of Artificial Intelligence and Electronic Engineering, Sichuan Technology and Business University, Meishan, Sichuan, China

**Keywords:** accident database, China, health hazards, LDA, LNG tank truck transportation, text mining, TF-IDF

## Abstract

LNG tank truck accidents can expose drivers, emergency responders, road users, and nearby populations to cryogenic release, fire, explosion, and oxygen-deficient environments, creating potentially serious health hazards. This study analyses 52 LNG tank truck transportation accident cases reported in China during 2015–2024 to examine whether a reproducible text-mining workflow can complement manual accident review and support health-hazard identification. The reports were segmented into 1,707 analytical text units, of which 1,393 valid units were retained after preprocessing. TF–IDF was used to identify discriminative terms and Latent Dirichlet Allocation was used to derive five latent themes, with topic selection based on perplexity, coherence, and semantic interpretability. Manual semantic validation was then used to distinguish statistical co-occurrence from conceptual or causal relationships. The resulting themes describe accident and response processes, human and management factors, external environmental conditions, equipment and facilities, and institutional and regulatory factors. Accident evidence was further interpreted in relation to documented or technically established pathways leading to frostbite, thermal burns, blast-related trauma, and asphyxiation. The findings demonstrate the value of combining reproducible text screening with expert interpretation for accident learning, health-hazard prevention, and risk management. The study does not estimate population-level health outcomes, health-economic effects, or the health effects of the low-carbon energy transition.

## 1 Introduction

LNG road transportation provides flexible gas supply where pipeline networks do not fully meet spatial, peak-demand, or emergency requirements. In China, LNG tank trucks are used for last-mile supply, peak-shaving, and emergency support. The continued use of LNG logistics creates an important occupational and community safety issue because transportation accidents may expose drivers, emergency responders, road users, and nearby residents to hazardous physical and chemical conditions. The low-carbon energy transition is therefore treated in this study as contextual motivation for the continuing operational relevance of LNG transport, rather than as an empirically tested determinant of health outcomes (1).

LNG tank truck transportation combines road-traffic exposure with hazardous-material properties. LNG is stored at approximately −162 °C near atmospheric pressure; loss of containment may cause rapid vaporization and dispersion and, when ignition conditions are present, fire or explosion scenarios. These events can generate health-relevant exposure pathways, including cryogenic contact, thermal radiation, blast effects, and oxygen displacement, with potential consequences such as frostbite, thermal burns, blast-related trauma, and asphyxiation. Accordingly, the public-health relevance of the present study lies in identifying and interpreting accident-related health hazards affecting exposed populations, rather than in estimating population-level disease burden or evaluating public-health policy outcomes.

As LNG road transportation has expanded rapidly, research has gradually shifted from descriptive accident investigations toward accident causation analysis, consequence modeling, and quantitative risk assessment. Existing studies have primarily focused on leakage probability, hazardous substance dispersion, thermal radiation, and explosion overpressure, whereas the associated health consequences have received comparatively less attention (2). Traditional methods, including Fault Tree Analysis (FTA) and Event Tree Analysis (ETA), have been extensively applied to identify accident causation pathways and logical relationships among accident events (3). More recently, computational fluid dynamics (CFD) and Bayesian Networks (BNs) have improved the simulation of accident propagation and supported dynamic risk inference (4). Although these approaches have substantially advanced the understanding of accident mechanisms and consequence evolution in LNG transportation, they rely mainly on physical models, experimental observations, or structured statistical data. Consequently, the rich unstructured information contained in accident investigation reports—including human behavior, equipment condition, environmental disturbances, management deficiencies, and emergency response activities—has not yet been fully exploited.

Accident investigation reports contain information on event sequences, direct and underlying causes, emergency actions, and reported casualties, and therefore provide a useful evidence base for accident learning (5). Database-based accident studies also demonstrate the value of systematically analyzing recurring patterns across hazardous-material and emerging-energy events (6). Automated text methods can improve consistency and auditability when narrative corpora are screened, but they do not replace contextual interpretation of human behavior, organizational deficiencies, or causal mechanisms (7). The present study therefore combines automated feature extraction and topic modeling with manual semantic validation.

Recent artificial-intelligence and data-driven safety studies provide complementary perspectives for extending the present text-mining framework. Work on AI-enabled environmental governance and sustainable industrial transformation highlights the broader value of intelligent decision support for risk governance (8, 9). Vision-language and multimodal domain-adaptation research suggests that future accident studies may integrate textual reports with images or other unstructured evidence (10). Spatial data-mining studies of crash hotspots show how geographical crash-pattern detection can complement narrative accident analysis (11, 12), while EEG-based driving-fatigue detection illustrates how text-derived human-factor themes may be linked with behavioral or physiological monitoring data (13).

Against this background, the central purpose of this study is to determine whether a reproducible TF–IDF–LDA workflow, combined with expert-guided semantic validation, can identify recurring risk themes in Chinese LNG tank truck accident narratives and support a structured interpretation of accident-related health-hazard pathways. The approach is intended to complement, rather than replace, manual qualitative accident analysis and to provide evidence relevant to accident prevention, emergency preparedness, and health protection.

The study makes three bounded contributions. First, it provides a transparent workflow for converting heterogeneous Chinese accident narratives into reproducible analytical text units and latent themes. Second, it distinguishes data-derived accident evidence from expert interpretation and from health-hazard mechanisms established in LNG consequence research, thereby preventing text-model output from being treated as direct epidemiological or causal evidence. Third, it translates the resulting evidence into practical implications for accident prevention, emergency preparedness, health protection, and risk management. These contributions are descriptive and methodological; they do not constitute estimates of population-level health outcomes, health-economic effects, or the health effects of the low-carbon transition.

**Figure 1:**
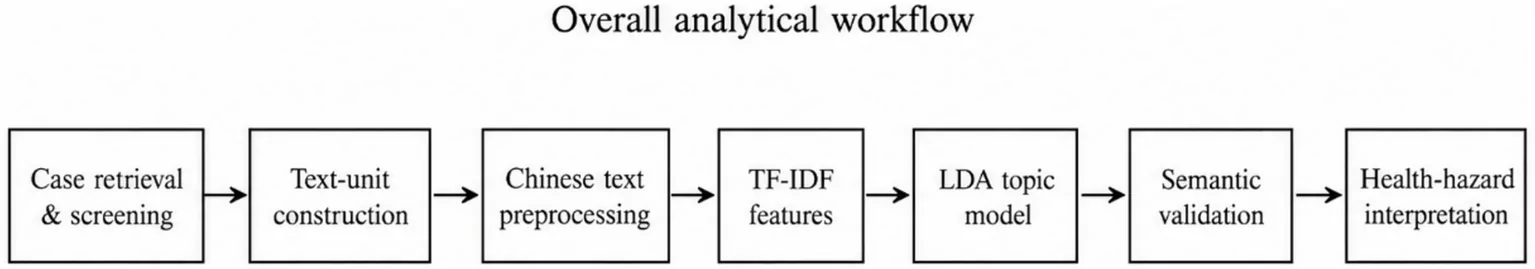
Overall workflow from accident-case retrieval to health-hazard interpretation.

The remainder of the article is organized as follows. Section 2 describes case retrieval, text-unit construction, and preprocessing. Section 3 presents TF–IDF and LDA procedures and results. Section 4 interprets the themes and develops the indicator framework. Section 5 distinguishes data-derived health evidence from technically inferred hazard pathways and discusses management implications. Section 6 concludes with limitations and future research directions (Figure 1).

## 2 Data sources and preprocessing

### 2.1 Data sources and sample selection

The primary sources were official accident investigation reports released by the Ministry of Emergency Management of the People’s Republic of China and provincial emergency-management authorities. Searches covered 2015–2024 and used combinations of Chinese terms equivalent to “LNG”, “liquefied natural gas”, “tank truck/tanker”, “road transportation”, “leakage”, “fire”, “explosion”, “collision”, and “rollover”. Cases were included when an LNG road tank vehicle was involved and sufficient information was available on the event sequence, causes, consequences, or emergency response. Duplicate reports, non-LNG hazardous-material events, stationary-facility events without a road-transport component, and records lacking sufficient narrative information were excluded. The final sample comprised 52 accident cases. “Representative” denotes coverage of multiple accident types and years; statistical representativeness of all LNG transport accidents is not claimed.

Where official reports contained limited contextual information, peer-reviewed articles, industry technical reports, and mainstream media were used only to cross-check dates, locations, event sequences, and emergency-response descriptions. The 52 cases are case-level observations. Each report was then divided into analytical text units, primarily semantically coherent sentences or short report segments describing one event, cause, response action, or consequence. This produced 1,707 text units (approximately 43,700 words). After duplicate, incomplete, and low-information units were removed, 1,393 valid units remained. Thus, 1,707 and 1,393 refer to text units, not separate accidents.

### 2.2 Text cleaning and standardization

Before analysis, the accident narratives were cleaned and standardized at the analytical-text-unit level. Duplicate units and units without substantive accident information were removed; event-sequence, causal, response, and consequence information was retained; dates, locations, vehicle descriptions, accident types, and casualty expressions were standardized; and each retained unit preserved its case identifier.

(1) *Redundancy removal*: Duplicate accident reports and records with substantial missing information were excluded to reduce sample duplication and potential bias.
(2) *Extraction of key information*: Information concerning accident sequences, causation analysis, emergency response, and corrective actions was extracted and organized into a structured format.
(3) *Variable standardization*: Key variables, including accident occurrence time, location, vehicle type, accident category, and casualty information, were coded using a unified scheme to improve data consistency and facilitate comparative analysis.
(4) *Corpus reconstruction*: The unstructured accident reports were reorganized into a standardized textual corpus suitable for subsequent word segmentation and topic modeling.

Following this procedure, 1,393 valid analytical text units were retained for further analysis.

### 2.3 Natural language processing and feature construction

The reconstructed Chinese corpus was processed using Jieba in accurate segmentation mode. A domain stop-word list combined common Chinese function words with corpus-specific non-informative expressions. A synonym dictionary merged equivalent forms, including vehicle-name variants and contextually equivalent accident expressions. Nouns, verbs, and selected adjectives carrying operational or consequence information were retained; punctuation, numerals without analytical meaning, isolated place names, and low-information tokens were excluded.

(1) *Word segmentation*: The Chinese accident reports were segmented into lexical tokens using the Jieba segmentation toolkit.
(2) *Stop-word removal*: Function words, frequently occurring general terms, and other expressions with limited semantic value were removed to reduce textual noise.
(3) *Synonym normalization*: Semantically equivalent expressions, such as *explosion* and *combustion explosion*, or *accident* and *incident*, were merged to improve semantic consistency throughout the accident corpus.
(4) *Part-of-speech filtering*: Informative lexical categories, particularly nouns and verbs, were retained, whereas redundant and low-information terms were excluded to improve the effectiveness of subsequent modeling.

After preprocessing, the final corpus comprised 1,393 valid analytical text units. A reproducibility log was maintained for segmentation, stop-word removal, synonym mapping, and part-of-speech filtering. Synonymous vehicle expressions were normalized to a common LNG tank-truck concept, while semantically distinct process terms such as leakage, ignition, rescue, and inspection were retained separately.

### 2.4 Determination of the optimal number of topics

Candidate LDA models with *k* = 2–10 topics were evaluated using the same preprocessed corpus and vocabulary. Model selection considered perplexity, topic coherence, semantic redundancy, and interpretability. The five-topic solution was retained because the largest reduction in perplexity occurred before *k* = 5, while additional topics produced only modest fit gains and increasingly fragmented or overlapping themes. A higher coherence value at larger *k* was therefore not treated as sufficient on its own to justify a more complex model.

**Figure 2:**
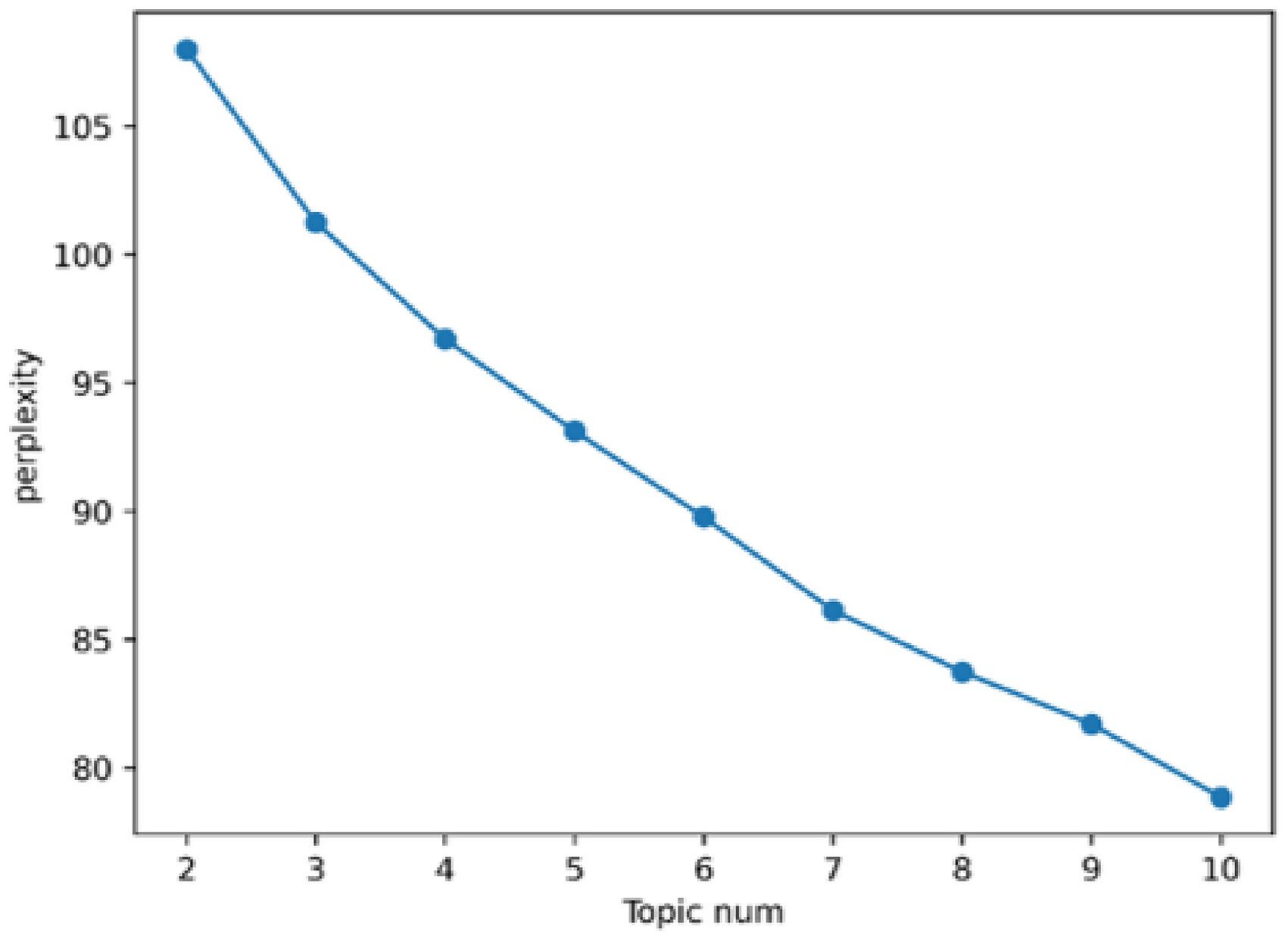
Perplexity as a function of the number of topics.

As shown in Figure 2, perplexity declined as the number of topics increased, reflecting an improvement in the model’s fit to the accident corpus. The most pronounced reduction occurred when the number of topics increased from 2 to 5, with perplexity decreasing from approximately 108 to 93. Beyond five topics, perplexity continued to decline, but the magnitude of improvement became considerably smaller. This pattern suggests that increasing the number of topics beyond five produced only limited additional gains in model fitting.

**Figure 3:**
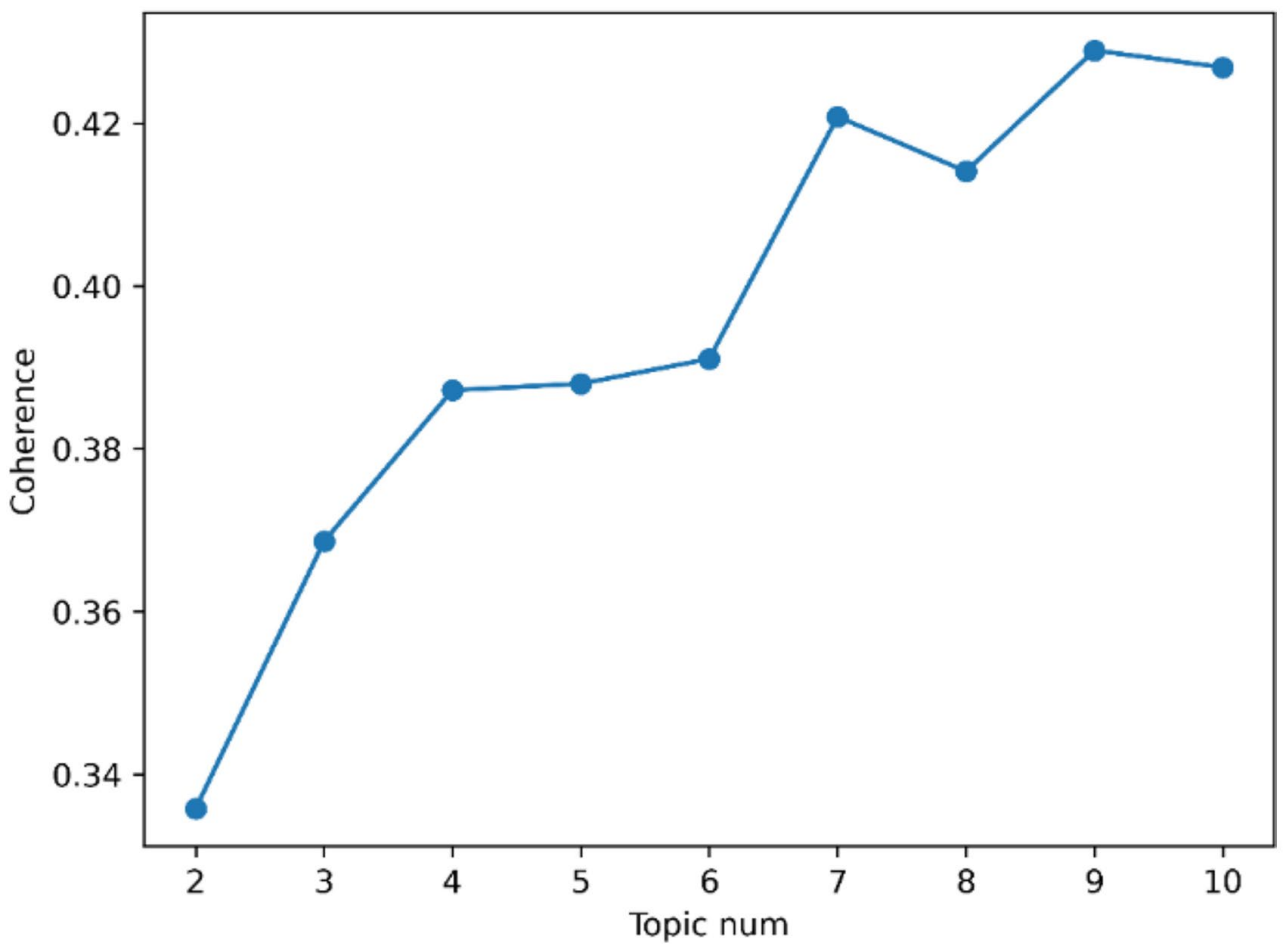
Topic coherence as a function of the number of topics.

The topic coherence results in Figure 3 show a rapid increase from approximately 0.34 to 0.39 as the number of topics rose from 2 to 4. Coherence subsequently increased at a slower rate and reached its highest value at nine topics, at approximately 0.43. However, models containing more than five topics produced greater semantic fragmentation and increased overlap among several topics. Although the numerical coherence score improved slightly, the distinctions among the extracted topics became less clear, which reduced their substantive interpretability.

Taken together, *k* = 5 was selected as a parsimonious compromise rather than as a uniquely optimal statistical solution. The decision reflects the perplexity elbow, acceptable coherence, and manual inspection of top-probability terms for semantic distinctiveness. Larger *k* values separated closely related accident vocabulary into narrower clusters without improving the conceptual usefulness of the themes.

## 3 Methodology and models

### 3.1 Methodology

#### 3.1.1 Principle of the TF–IDF method

Term Frequency–Inverse Document Frequency (TF–IDF) is a widely used keyword extraction method in text mining. It evaluates the importance of a term by considering both its frequency within a particular document and its distribution across the entire corpus. A term that appears frequently in one document but occurs less frequently in the remaining documents is generally considered more representative of the distinctive content of that document.

Term frequency (TF) reflects the importance of a term within an individual document and is measured by the frequency with which term w occurs in document d . Inverse document frequency (IDF), by contrast, measures the ability of a term to distinguish among documents according to its distribution across the corpus. Terms appearing in a large number of documents receive relatively low IDF values, whereas terms occurring in fewer documents are assigned to higher weights. The IDF of term w is calculated using Equation (1):


$$\mathrm{IDF}(w)=\log\frac{N}{\mathrm{df}(w)}+1$$ (1)

Where N denotes the total number of documents in the corpus, and df(w) represents the number of documents containing term w .

The TF–IDF weight of term w in document d is obtained by multiplying its t erm frequency by its inverse document frequency, as shown in Equation (2):


TF−IDF(w,d)=TF(w,d)×IDF(w) (2)

In the present analysis, the extracted high-weight keywords provided the lexical basis for LDA topic modeling and supported the identification of latent risk themes in LNG tank truck transportation accidents.

#### 3.1.2 Application of the TF–IDF method to accident text analysis

The TF–IDF algorithm was applied to extract representative textual features from LNG tank truck accident reports. Its application served three main purposes:

(1) to identify high-weight domain-specific terms, such as *leakage*, *valve*, and *explosion*, that reflect the principal characteristics of accident events;
(2) to reduce the influence of common words with limited semantic value, thereby minimizing textual noise and improving feature representation;
(3) to construct a Bag-of-Words (BoW) representation for LDA topic modeling.

### 3.2 Word frequency and keyword analysis

Word frequency analysis was conducted to examine the distribution of core terms within the accident corpus and to identify the vocabulary that most frequently appeared in LNG tank truck transportation accidents. Following text preprocessing, including word segmentation, stop-word removal, and synonym normalization, term frequencies were calculated using the corpus of 52 representative LNG tank truck accident reports collected in China between 2015 and 2024. The high-frequency keywords identified from the accident corpus are summarized in Table 1.

**Table 1:** High-frequency keywords identified from LNG tank truck accident texts.

Rank	Keyword	Frequency	Description
1	Accident	239	Represents the central theme of the accident corpus, referring to accident events themselves and reflecting the primary focus of the study.
2	Scene	184	Indicates that accident reports predominantly describe on-site emergency response and accident management activities.
3	Natural gas	179	Identifies the hazardous substance involved, highlighting the energy-related characteristics and hazardous properties of LNG.
4	Leakage	173	Represents one of the most common initiating accident events, frequently leading to secondary consequences such as fires and explosions.
5	Occurrence	165	Relates to the occurrence of accident events, emphasizing the sudden and unexpected nature of accident development.
6	Safety	132	Highlights the critical role of safety management, regulatory measures, and risk prevention in accident control.
7	Rescue	118	Reflects the central importance of emergency rescue operations throughout accident response and mitigation processes.
8	Firefighting	106	Indicates the involvement of professional firefighting forces in emergency response and accident control.
9	Emergency response	91	Refers to the emergency measures and response actions implemented following accident occurrence to mitigate accident consequences.

The accident corpus was dominated by terms closely associated with accident events and emergency response. As shown in Table 1, “accident” (239 occurrences), “scene” (184), “natural gas” (179), “leakage” (173), and “occurrence” (165) were among the most frequently occurring terms. Their prevalence reflects the emphasis of accident investigation reports on accident development and emergency situations during LNG tank truck transportation.

Terms associated with emergency response and safety management also appeared frequently, including “safety” (132), “rescue” (118), “firefighting” (106), and “emergency response” (91). These high-frequency terms demonstrate that emergency management and safety assurance constitute important components of LNG transportation accident investigations.

Although “accident” appeared most frequently in the corpus, its TF–IDF weight remained relatively low because the term occurred in the majority of documents and therefore contributed limited discriminative information. By comparison, domain-specific terms such as “LNG tank truck”, “leakage”, and “natural gas” exhibited both high frequencies and high TF–IDF weights. These terms more effectively captured the distinctive characteristics of LNG hazardous materials transportation and consequently provided a more informative lexical basis for subsequent LDA topic modeling and risk theme identification.

To further identify representative domain-specific features, TF–IDF keyword extraction was performed using the Jieba text mining toolkit. The topK parameter was set to 20, and the 20 highest-ranked keywords were extracted from each accident report according to their TF–IDF weights. The resulting keywords and their corresponding weights are summarized in Table 2, providing a quantitative description of the most informative lexical features within the accident corpus.

**Table 2:** High-weight keywords and their TF–IDF scores in LNG tank truck accident texts.

Rank	Keyword	TF–IDF score	Rank	Keyword	TF–IDF score
1	LNG tank truck	0.2718	11	Truck	0.0803
2	Accident	0.2562	12	Personnel	0.0790
3	Natural gas	0.1805	13	Authorities	0.0636
4	Scene	0.1747	14	Zibo city	0.0610
5	Firefighting	0.1474	15	Butyl	0.0576
6	Vehicle	0.1222	16	Fire engine	0.0539
7	Tank truck	0.1082	17	Peroxide	0.0477
8	Hazardous chemicals	0.1061	18	Responsibility	0.0473
9	Tanker truck	0.1042	19	Emergency command headquarters	0.0464
10	Tank	0.0865	20	Qualification	0.0418

High-weight terms, including “LNG tank truck”, “accident”, “natural gas”, and “scene”, were closely associated with the transportation vehicle, accident evolution, and emergency response processes, capturing the principal characteristics of LNG transportation accidents. By comparison, management-related terms such as “responsibility”, “qualification”, and “authority” received relatively lower TF–IDF weights. Although these terms appeared less frequently among the highest-weight keywords, they point to underlying institutional and organizational issues related to regulatory compliance, operational qualifications, and accountability. This pattern suggests that deficiencies in safety management and governance remain important contributing factors to accident occurrence.

### 3.3 Construction of the LDA topic model

#### 3.3.1 Determination of the number of topics (*k*)

The number of LDA topics (*k*) is a model parameter and should not be confused with the number of keywords shown in frequency or TF–IDF tables. *k* = 5 means that five latent topic distributions were estimated; each topic is characterized by multiple high-probability terms. Table 1 therefore reports corpus-level high-frequency terms rather than five topics.

For reproducibility, LDA was fitted to the 1,393 analytical text units using the same dictionary and bag-of-words corpus for each candidate *k* from 2 to 10. Symmetric priors were used as the baseline specification, and the random seed was fixed for repeatable comparison. Perplexity and coherence were treated as diagnostics, while final selection also required manual semantic inspection for fragmentation and overlap. Perplexity was calculated using Equation (3):


$$\text{Perplexity}=\exp(-\frac{\sum_{d=1}^{D}\log\;p\;(d)}{w})$$ (3)

where D denotes the total number of documents in the corpus, W represents the total number of terms, and p(d) is the likelihood of document d.

Figures 2 and 3 show that perplexity decreased steadily as the number of topics increased, indicating an improved fit to the accident corpus. The reduction became much smaller once *k* = 5, suggesting limited additional improvement from introducing more topics. Topic coherence also increased with the number of topics and reached a relatively high level around *k* = 5. Although slightly higher coherence values were obtained for larger topic numbers, several extracted topics became increasingly fragmented and exhibited greater semantic overlap, making their interpretation less straightforward.

Taken together, these evaluation results support the selection of *k* = 5 as the optimal topic number. This configuration provides an appropriate balance between statistical fitness and semantic interpretability while capturing the principal risk structure embedded in the accident corpus. All subsequent analyses of risk theme identification and topic interpretation were therefore based on the five-topic model.

#### 3.3.2 Topic identification and topic weights

The LDA model generated five latent word distributions. Topic labels were assigned only after manual review of the highest-probability terms and their source text units. Automated co-occurrence was not treated as evidence that all terms belonged to the same causal stage. In particular, the Accident and Response Process Theme contains vocabulary from both accident occurrence and post-event response; these stages are kept conceptually distinct in interpretation even though they co-occurred within the same latent topic.

The average to pic weight was calculated using Equation (4):


$$\theta_{k}=\frac{1}{N}\sum_{d=1}^{N}P(z=k|d)$$ (4)

where $\theta_{k}$ denotes the average weight of topic (*k*), [ P(z=k∣d) ] represents the probability of topic (k) in document (d), and (N) is the total number of documents in the corpus.

The five identified themes differed considerably in their relative prevalence across the accident corpus, revealing the multidimensional nature of accident risks in LNG tank truck transportation. The characteristics of each theme are summarized below.

(1) Accident and Response Process Theme (26.5%)

This theme accounted for the largest proportion of the accident corpus. Representative keywords included “leakage”, “rear-end collision”, “fire”, “firefighting”, and “rescue”. These terms characterize accident initiation together with the subsequent emergency response process. From the perspective of accident evolution, this theme mainly corresponds to the initial triggering–immediate response stage, emphasizing that accident occurrence and emergency intervention remain the dominant concerns in LNG tank truck transportation safety.

(2) Human and Safety-Management Theme (23.1%)

With a topic weight of 23.1%, this theme ranked second among the identified risk categories. Keywords such as “unsafe operation”, “fatigue driving”, “safety training”, and “accountability” underline the influence of human behavior and management practices on accident occurrence. Rather than arising from external disturbances, these risks are primarily associated with operational deviations and deficiencies in the implementation of safety management systems, both of which increase the likelihood of accidents.

(3) External Environment Theme (19.2%)

Representative keywords, including “road conditions”, “weather conditions”, and “traffic congestion”, describe the influence of external environmental conditions on transportation safety. These factors introduce substantial uncertainty into the transportation process and are generally difficult to control directly. Consequently, they represent an important external source of accident risk in LNG tank truck transportation.

(4) Equipment and Facility Theme (18.2%)

This theme is characterized by terms such as “tank”, “valve”, “inspection”, and “maintenance”, reflecting the condition and reliability of transportation equipment. Equipment degradation or inadequate maintenance may allow latent defects to accumulate over time, eventually developing into observable accident events when combined with adverse operating conditions.

(5) Institutional and Regulatory Theme (13.0%)

Although this theme accounted for the smallest topic weight, keywords including “supervision”, “law enforcement”, and “regulatory implementation” highlight the importance of institutional governance in accident prevention. Unlike the preceding themes, these factors generally influence accident occurrence indirectly by shaping regulatory compliance and the implementation of safety management practices. They therefore represent background risk factors that support the long-term safety of LNG tank truck transportation.

### 3.4 Construction of the risk Indicator system

The five LDA themes were mapped to a structured indicator framework through a second-stage qualitative interpretation. The mapping was informed by accident narratives, established hazardous-material transport concepts, and engineering judgment. The indicator system should therefore be understood as an analytical framework derived from, but not fully measured by, the 52 cases. Indicators directly observable in many reports (e.g., collision type, leakage occurrence, road/weather condition, or reported response actions) were distinguished from proposed operational indicators requiring enterprise or regulatory data (e.g., training coverage, inspection frequency, maintenance interval, and response-time performance).

### 3.5 Topic visualization analysis

#### 3.5.1 Visualization of risk theme distribution and relationships

**Figure 4:**
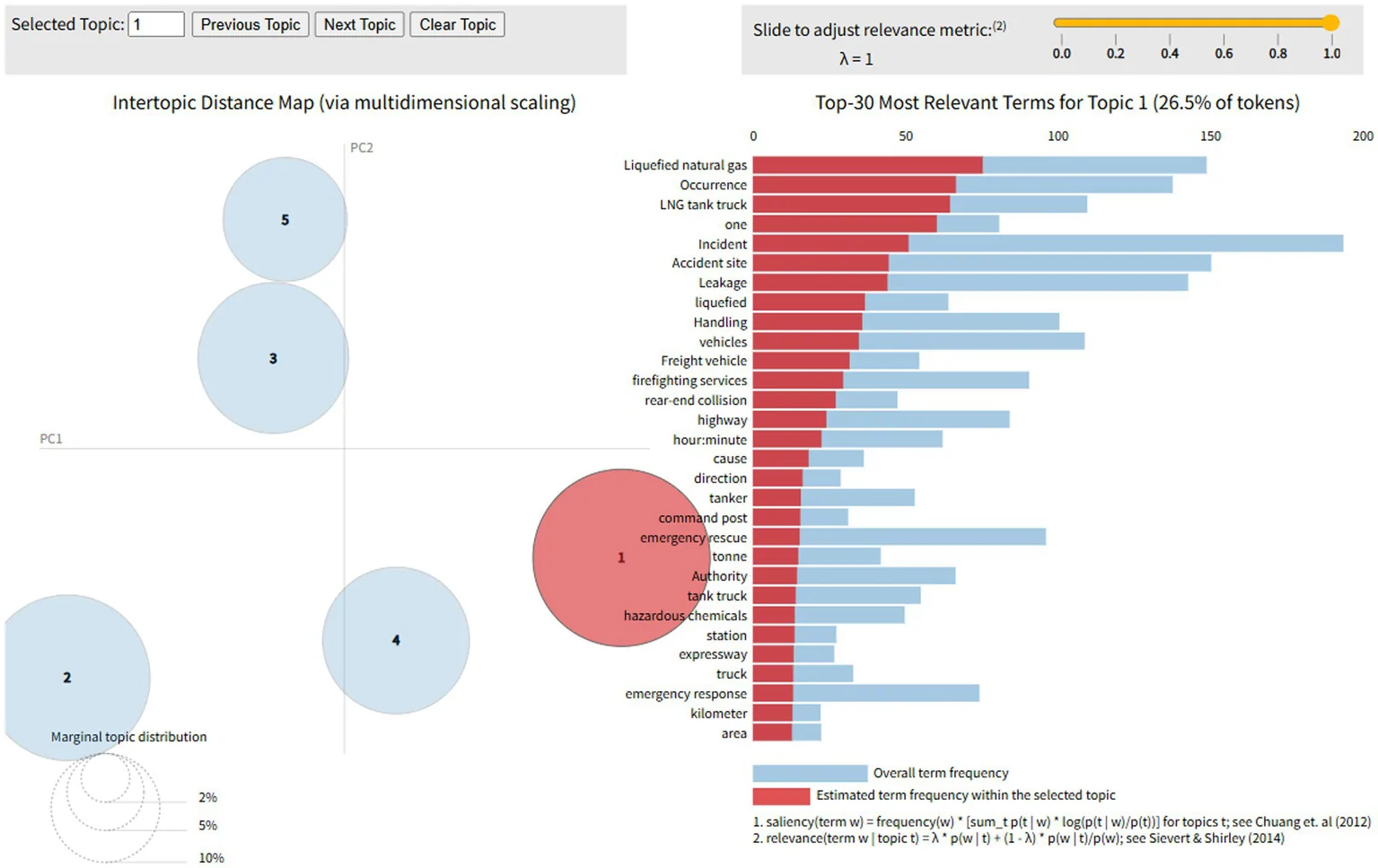
LDA intertopic-distance view for the Accident and Response Process Theme; Bubble size represents topic prevalence and term bars show the most relevant words. Topic 1: Accident and Response Process Theme (Average Topic Weight: 26.5%).

**Figure 5:**
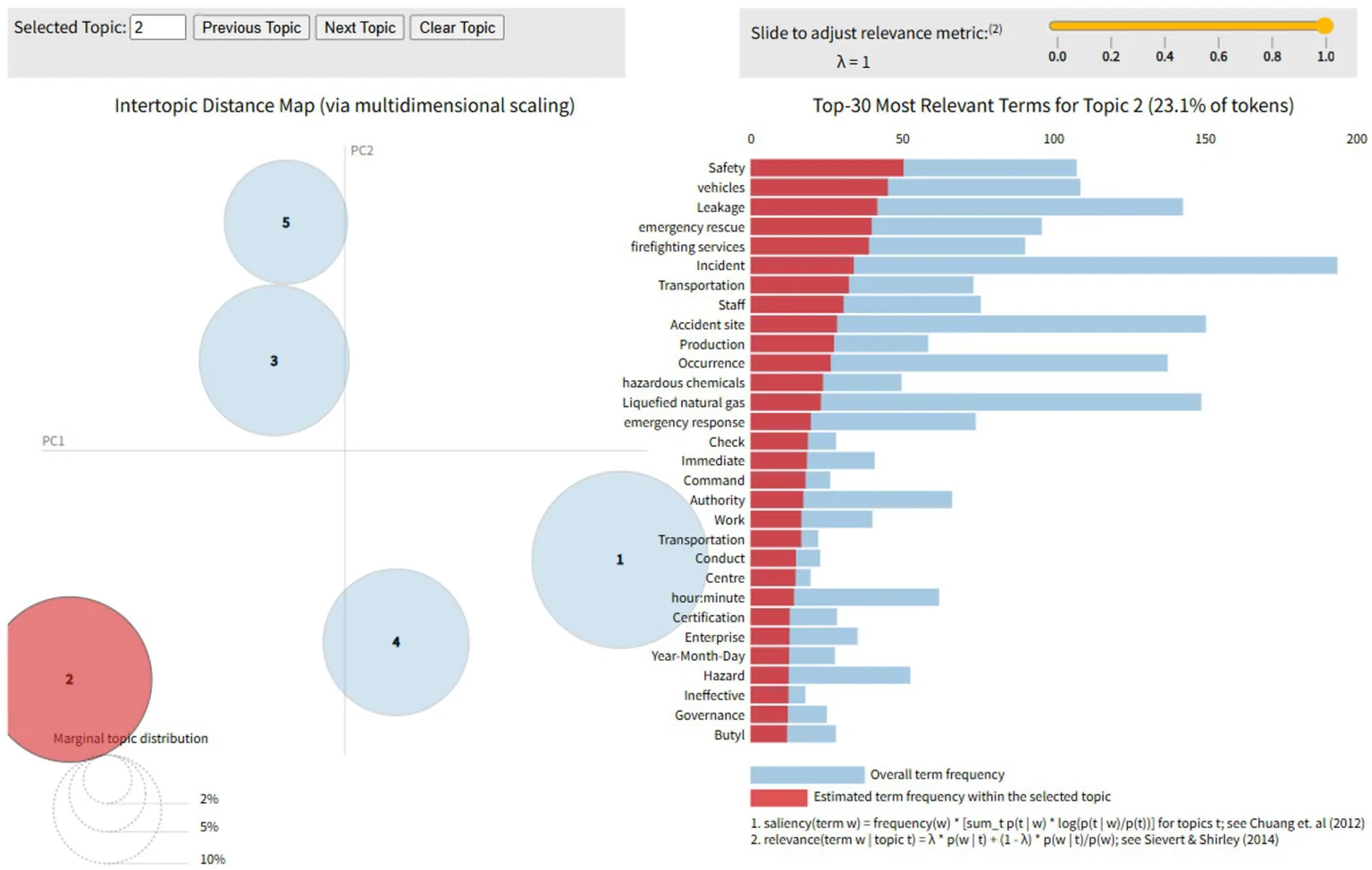
LDA intertopic-distance view for the Human and Safety-Management Theme; labels and term relevance are presented at enlarged scale. Topic 2: Human and Safety-Management Theme (Average Topic Weight: 23.1%).

**Figure 6:**
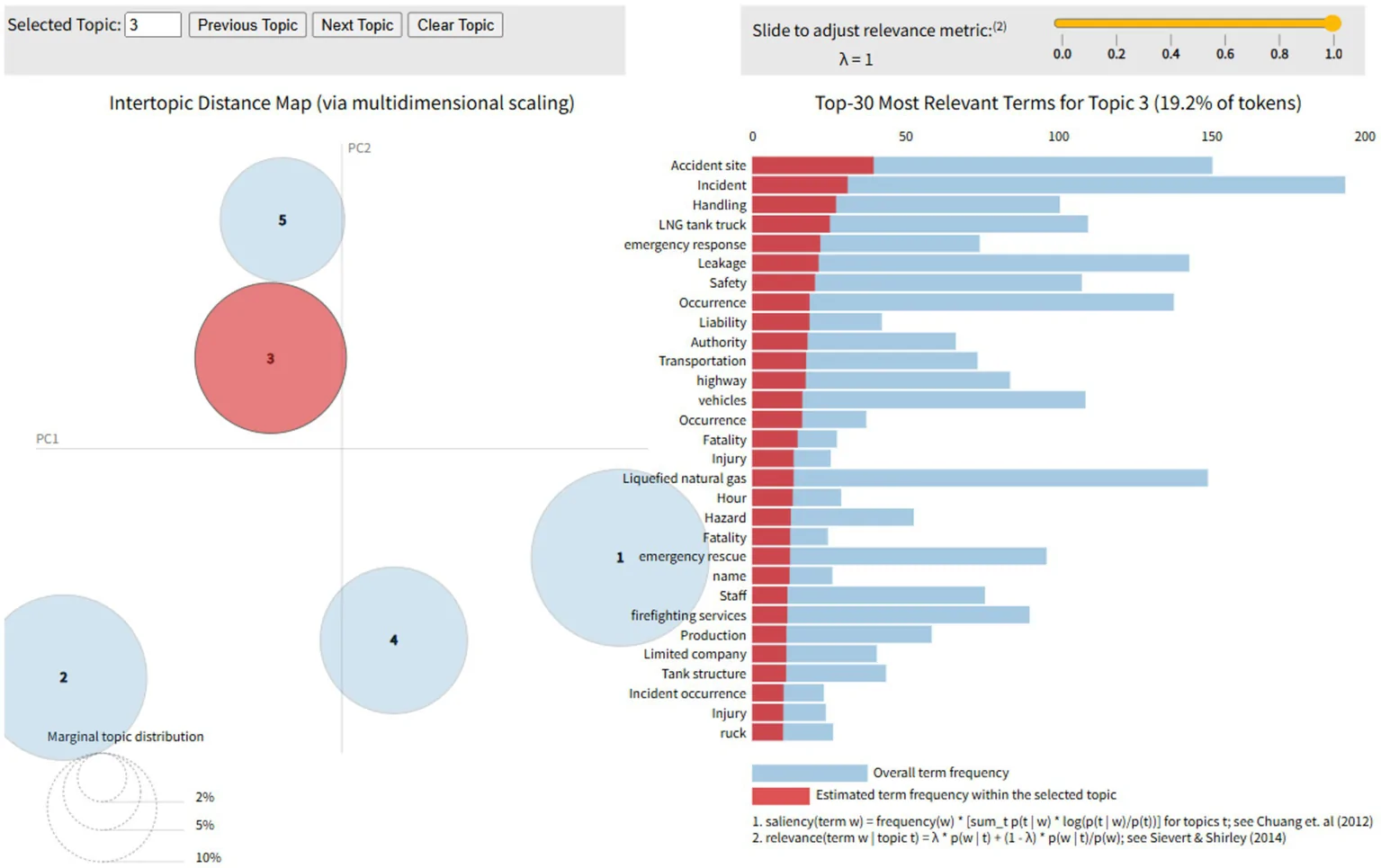
LDA intertopic-distance view for the External Environment Theme; spatial separation indicates semantic distinctiveness from other themes. Topic 3: External Environment Theme (Average Topic Weight: 19.2%).

**Figure 7:**
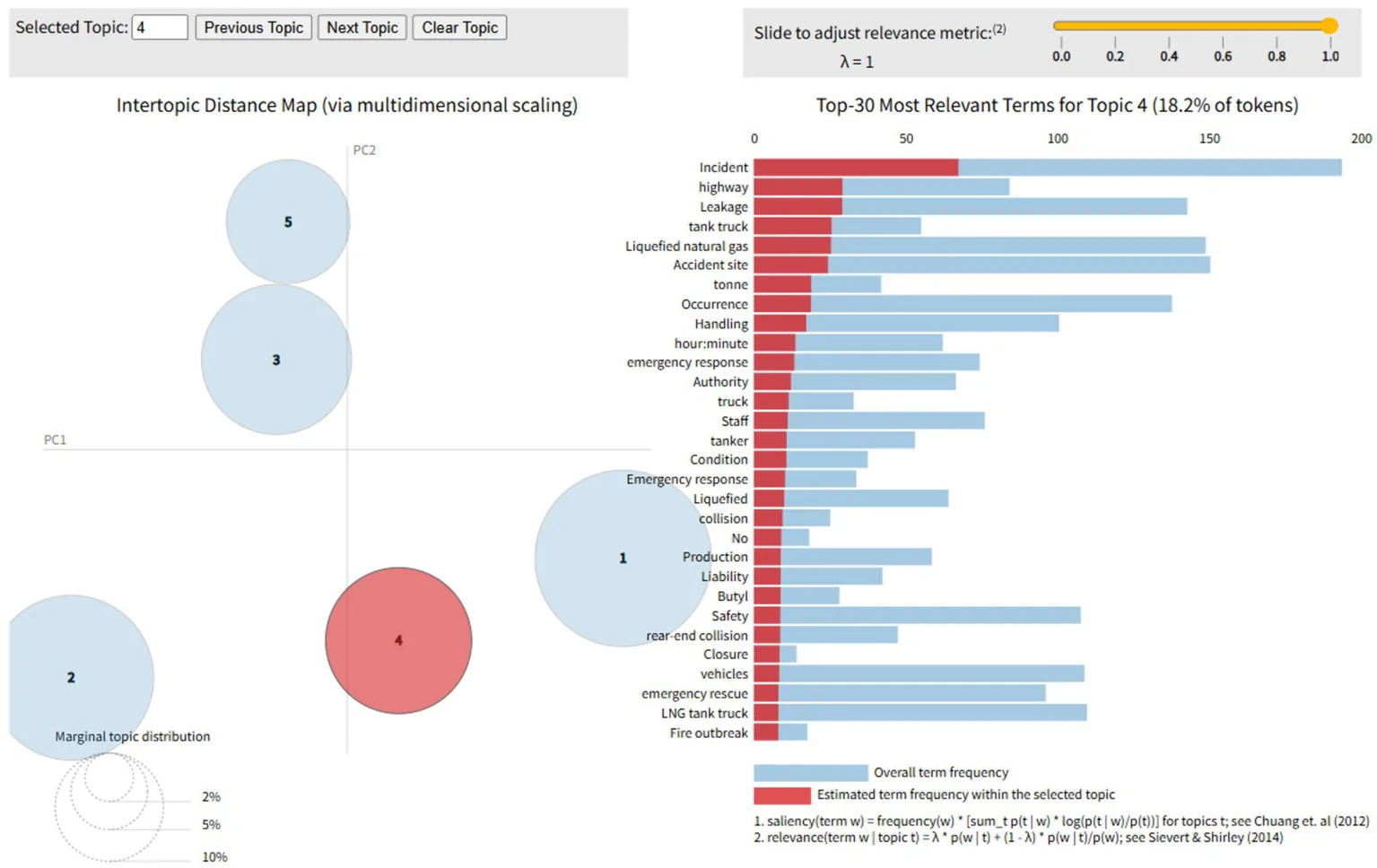
LDA intertopic-distance view for the Equipment and Facility Theme; representative terms relate to tanks, valves, inspection, and maintenance. Topic 4: Equipment and Facility Theme (Average Topic Weight: 18.2%).

**Figure 8:**
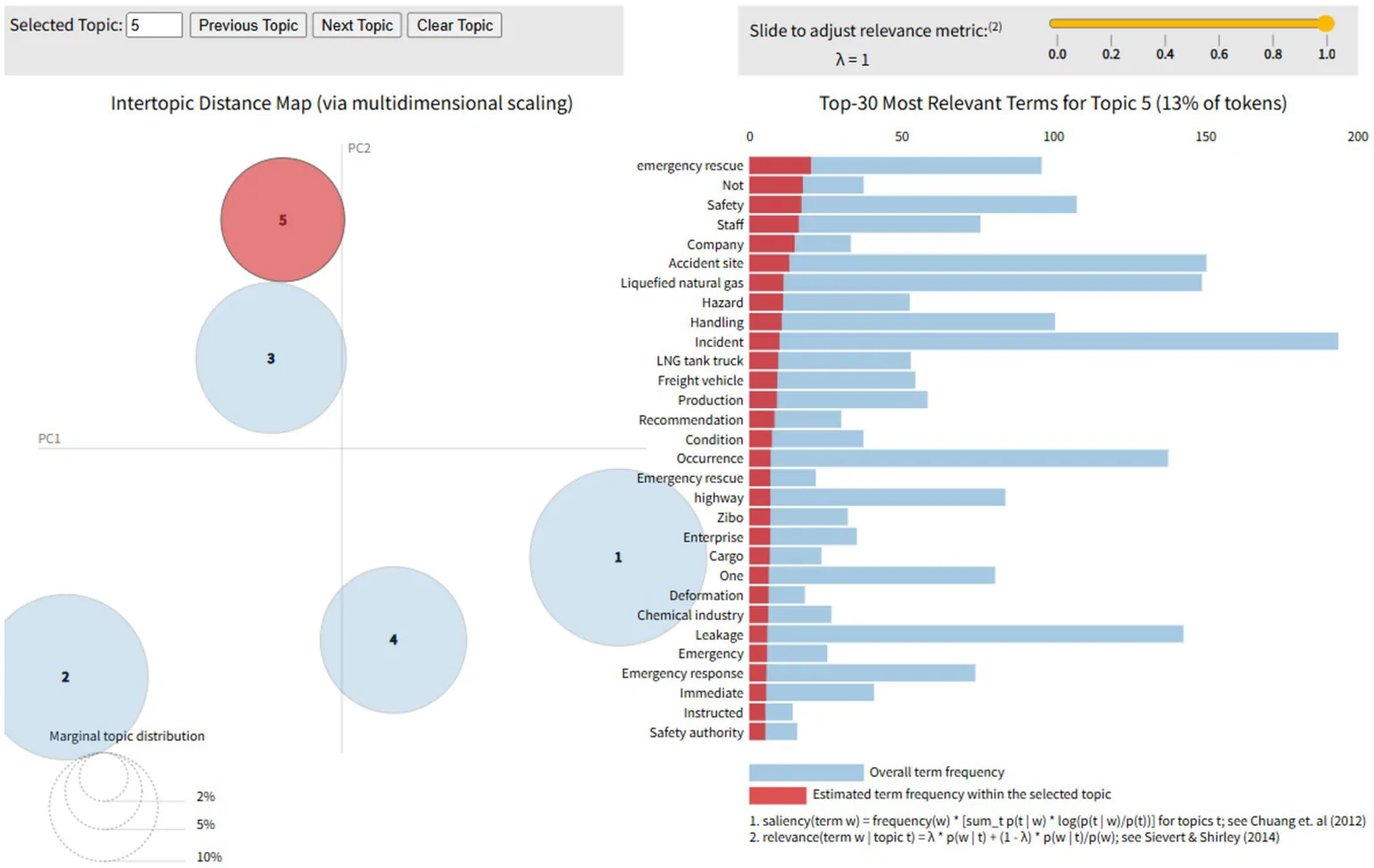
LDA intertopic-distance view for the Institutional and Regulatory Theme; representative terms concern supervision, qualification, compliance, and enforcement. Topic 5: Institutional and Regulatory Theme (Average Topic Weight: 13.0%).

To examine the relationships among the identified risk themes, an intertopic distance map generated by the LDA model was used to visualize their distribution within the accident corpus (Figures 4–8). In the visualization, the size of each bubble represents the corresponding topic weight, different colors distinguish individual risk themes, and the distance between bubbles reflects their semantic similarity. Visualization shows clear differences in the relative prominence of the identified themes. Topics characterized by keywords associated with natural gas leakage and accident rescue are represented by relatively large bubbles, indicating that these risk themes account for a substantial proportion of the accident corpus. By comparison, topics dominated by keywords related to tank truck leakage and enterprise qualification occupy smaller regions in the semantic space, suggesting that these themes occur less frequently and are more closely associated with specific accident scenarios or industry contexts (14).

#### 3.5.2 Identification of risk themes

The TF–IDF keyword extraction results and LDA topic modeling were jointly interpreted to examine the risk characteristics of 52 LNG tank truck transportation accident reports collected in China between 2015 and 2024. High-frequency keywords, together with the semantic features of each topic, were used to characterize and distinguish the principal accident risk patterns identified in the accident corpus (15). The analysis yielded five major risk themes: Accident and Response Process Theme, Human and Safety-Management Theme, External Environment Theme, Equipment and Facility Theme, and Institutional and Regulatory Theme. Collectively, these themes describe the multidimensional risk characteristics of LNG tank truck transportation accidents and establish a structured analytical framework for subsequent risk-management analysis and practical implications.

The five identified risk themes collectively capture the major stages of accident occurrence, accident evolution, and emergency response while representing the influence of human, equipment, environmental, and institutional factors. Together, they provide a structured understanding of the principal sources of risk in LNG tank truck transportation accidents and establish the analytical basis for subsequent health hazard and risk governance analyses.

## 4 Interpretation of accident themes and construction of a risk-Indicator framework

Terminology is used consistently as follows. A hazard is a source or situation with the potential to cause harm; risk concerns the possibility and consequences of harm under specified conditions; hazard identification concerns recognition of hazardous sources or scenarios; and risk assessment requires explicit evaluation of likelihood and consequence. Because this study does not estimate case-specific probabilities for all indicators, Section 4 is framed as theme interpretation and indicator construction rather than as a complete quantitative risk assessment.

### 4.1 Interpretation of risk themes

The LDA topic model identified five major risk themes that characterize the principal dimensions of risk in LNG tank truck transportation accidents: Accident and Response Process Theme, Human and Safety-Management Theme, External Environment Theme, Equipment and Facility Theme, and Institutional and Regulatory Theme. Collectively, these themes capture the major stages of accident occurrence, accident evolution, and emergency response while reflecting the combined influence of human behavior, equipment condition, operating environment, and institutional factors. This statement summarizes findings generated by the present analysis and is therefore presented without an external citation. Comparison with prior studies is provided separately below.

Substantial variation was observed in the relative contribution of individual risk themes to the accident corpus. Accident and Response Process Theme (26.5%) and Human and Safety-Management Theme (23.1%) accounted for nearly half of the overall topic weight (49.6%), while External Environment Theme (19.2%) and Equipment and Facility Theme (18.2%) together accounted for 37.4%. This pattern is broadly consistent with large-scale hazardous-material road-accident research in China, which identifies human factors, vehicle conditions, road/environmental conditions, leakage, collision, and rollover as recurrent contributors or consequences (16). However, the present corpus additionally gives explicit analytical prominence to emergency-response processes and institutional/regulatory factors because accident investigation narratives contain post-event response and accountability information that is not always represented as a separate dimension in structured crash databases.

Taken together, the five risk themes describe the overall risk structure of LNG tank truck transportation accidents and establish the analytical basis for subsequent risk indicator extraction and health hazard identification.

(1) Accident and Response Process Theme (Topic 1, 26.5%)

Accident and Response Process Theme represented the largest component of the accident corpus, accounting for 26.5% of the overall topic weight. High-weight keywords, including natural gas (0.0196), LNG tank truck (0.0168), accident (0.0133), leakage (0.0115), and firefighting (0.0077), describe the progression from accident initiation to emergency response. Analysis of the accident reports shows that vehicle collisions, rollovers, and equipment damage frequently trigger natural gas leakage, which may subsequently escalate into fires or explosions. The effectiveness of emergency response plays a critical role in limiting accident propagation and reducing the severity of health hazards. Accordingly, accident type, accident severity, natural gas leakage volume, emergency response time, and on-site emergency response implementation rate were identified as the key risk indicators representing this theme.

(2) Human and Safety-Management Theme (Topic 2, 23.1%)

Ranked second with a topic weight of 23.1%, Human and Safety-Management Theme primarily describes the influence of driver behavior and organizational safety management on accident occurrence. Representative keywords include accident (0.0256), expressway (0.0111), leakage (0.0111), LNG tank truck (0.0097), and responsibility (0.0030). In particular, the occurrence of “expressway” and “responsibility” highlights the close relationship between driving practices, safety management, and transportation risk. Evidence from the accident corpus suggests that fatigue driving, speeding, improper operations, inadequate safety training, and insufficient implementation of safety responsibilities are recurring contributors to accident occurrence. Unlike environmental or equipment-related risks, these factors are largely controllable through standardized operating procedures, systematic safety training, and effective management practices. Based on these findings, driver violations, operational errors, safety training coverage, implementation of safety management systems, and the degree of responsibility implementation were selected as the key risk indicators for this theme.

(3) External Environment Theme (Topic 3, 19.2%)

Representative keywords for External Environment Theme include safety (0.0151), vehicle (0.0135), leakage (0.0125), rescue (0.0119), and transportation (0.0050). These keywords indicate that external operating conditions influence not only vehicle operational safety but also the effectiveness of emergency response. Overall, this theme accounted for 19.2% of the total topic weight. LNG tank truck transportation often involves long-distance operations across complex geographical environments. Mountainous roads, continuous curves, steep gradients, adverse weather conditions, and traffic congestion increase driving difficulty and may also delay emergency response following an accident. Such environmental disturbances are largely external to transportation operations but can substantially amplify accident consequences once an incident occurs. On this basis, road technical grade, traffic flow density, meteorological conditions, and the frequency of natural hazards were selected as the key risk indicators for evaluating External Environment Theme.

(4) Institutional and Regulatory Theme (Topic 5, 13.0%)

Although the Institutional and Regulatory Theme contributed the smallest topic weight (13.0%), its influence extends throughout transportation organization, enterprise safety management, and government supervision. High-weight keywords—including rescue (0.0108), safety (0.0091), company (0.0079), and Safety Supervision Bureau (0.0030)—highlight the importance of regulatory oversight and organizational responsibility in maintaining transportation safety. Unlike operational or equipment-related risks, institutional factors rarely trigger accidents directly. Instead, they influence accident prevention by strengthening transportation qualification management, improving safety management systems, enhancing hazard identification and risk inspection, and establishing comprehensive emergency response plans (17). These characteristics support the selection of enterprise qualification level, frequency of regulatory inspections, implementation of safety regulations, risk assessment systems, and emergency response plan coverage as the key risk indicators for this theme.

(5) Equipment and Facility Theme (Topic 4, 18.2%)

The Equipment and Facility Theme accounted for 18.2% of the overall topic weight and describes the contribution of transportation equipment and safety facilities to accident occurrence. Representative keywords include site (0.0143), LNG tank truck (0.0091), transportation (0.0063), leakage (0.0078), and tank (0.0040), covering vehicle components, storage and transportation equipment, and associated safety facilities. Evidence from the accident corpus suggests that tank damage, safety valve failure, loose pipeline connections, and inadequate vehicle maintenance are recurrent causes of natural gas leakage and may subsequently develop into secondary accidents such as fires and explosions. Because equipment reliability directly determines the safe containment and transportation of LNG, vehicle technical condition, equipment inspection and maintenance intervals, integrity of safety accessories, equipment abnormality records, and the level of facility safety assurance were identified as the key risk indicators representing this theme.

### 4.2 Analysis of risk structure characteristics

The results of the LDA topic model indicate that the risks associated with LNG tank truck transportation accidents are not caused by a single factor but arise from the combined effects of multiple factors, including human factors, equipment and facilities, the external environment, accident evolution, and institutional management. The five identified risk themes perform different functions during accident formation and exhibit distinct characteristics of hierarchy, coupling, and dynamic evolution.

#### 4.2.1 Human and safety-management factors are prominent in the accident corpus

According to the distribution of topic weights, Accident and Response Process Theme (26.5%) and Human and Safety-Management Theme (23.1%) ranked first and second, respectively, accounting for a combined 49.6% of the total topic weight, which is nearly half of all identified risk themes. Accident and Response Process Theme reflect the critical stages of accident initiation, accident propagation, and emergency response, whereas Human and Safety-Management Theme extend throughout the entire transportation process, including driving behavior, safety management, and the implementation of safety responsibilities. The relatively high topic weights of these two risk themes indicate that human factors are not only major contributors to accident occurrence but also important determinants of accident consequences. It is noteworthy that human factors rarely act independently and usually interact with equipment conditions and the operating environment to influence accident occurrence. When driving errors coincide with equipment failures or complex road conditions, both the probability of accident occurrence and the severity of accident consequences increase significantly. Therefore, human factors primarily act as the direct triggers of accidents, whereas equipment and environmental factors largely determine the extent of accident development and the scope of accident impacts.

#### 4.2.2 Equipment and facilities together with the external environment constitute the fundamental conditions for accident occurrence

Equipment and Facility Theme (18.2%) and External Environment Theme (19.2%) exhibited similar topic weights, accounting for a combined 37.4% of the total topic weight. This finding highlights the important influence of transportation equipment reliability and the operating environment on transportation safety. Equipment and Facility Theme primarily involve the technical condition of vehicles, the structural integrity of LNG tanks, and the operating status of safety accessories. The reliability of these components is directly associated with the risk of natural gas leakage and the occurrence of secondary accidents. The External Environment Theme mainly arises from objective factors such as road conditions, traffic flow, meteorological conditions, and natural hazards. These factors not only affect the operational stability of vehicles but also directly constrain the efficiency of emergency response following an accident. In mountainous regions, high-altitude areas, and other complex terrains, continuous downhill grades, sharp curves, and adverse weather conditions further increase transportation risks. Therefore, the Equipment and Facility Theme and External Environment Theme represent important technical and environmental dimensions of LNG tank truck transportation risk and may influence both accident development and consequence severity.

#### 4.2.3 The institutional and regulatory theme provides safeguards throughout the entire transportation process

Institutional and Regulatory Theme accounted for 13.0% of the total topic weight. Although this risk theme had the lowest topic weight, its primary role lies in accident prevention and safety management, exhibiting long-term and fundamental characteristics. Institutional arrangements, including enterprise safety management systems, transportation qualification management, regulatory inspections, and emergency response plans, can provide continuous constraints on human operations, equipment operation, and emergency response through standardized transportation organization, safety training, equipment maintenance, and risk inspection and hazard identification. Therefore, although the Institutional and Regulatory Theme is not a direct trigger of accidents, it provides essential support for ensuring transportation safety and reducing accident risks.

#### 4.2.4 Risk factors exhibit distinct coupling and dynamic evolution characteristics

The five identified risk themes are not mutually independent but instead form a continuous chain of risk evolution throughout the transportation process. Human operational errors may trigger equipment failures, which can subsequently lead to natural gas leakage. Complex environmental conditions may further accelerate accident propagation, whereas emergency response capability and the effectiveness of institutional and regulatory measures directly influence accident control and the final consequences. Accordingly, LNG tank truck transportation accidents generally undergo a dynamic evolution process consisting of risk initiation, accident occurrence, accident propagation, emergency response, and accident control. Different risk factors play dominant roles at different stages of this process and jointly determine the trajectory of accident development through their interactions and coupling effects. These findings indicate that risk prevention and control for LNG tank truck transportation should be implemented in an integrated manner across multiple dimensions, including human factors, equipment and facilities, the external environment, and institutional and regulatory measures, rather than focusing on the management of any single risk factor.

#### 4.2.5 Implications of risk structure analysis for subsequent research

Risk structure analysis further clarifies the roles and relationships of different risk factors during the process of accident formation, thereby providing a theoretical basis for risk indicator extraction and the construction of a risk indicator system. The five risk themes identified from the accident corpus enable the transformation of unstructured textual information into structured risk indicators. In addition, these findings provide a foundation for subsequent studies on health hazard identification, risk assessment, and risk governance.

### 4.3 Risk Indicator extraction and construction of the risk Indicator system

The risk themes identified through LDA topic modeling provide a semantic representation of the major characteristics of LNG tank truck transportation accidents. However, semantic themes alone cannot be directly incorporated into quantitative risk assessment or health hazard analysis. To enable subsequent evaluation, the semantic information extracted from the accident corpus was translated into structured risk indicators, forming the basis for a comprehensive risk indicator system and supporting subsequent studies on health hazard identification and risk governance (18).

The risk indicator system was developed by integrating the results of accident text mining with accident investigation reports, regulations governing the road transportation of dangerous goods, and safety management requirements for LNG transportation. Through semantic analysis of the topic keywords, representative risk factors were identified and subsequently transformed into structured risk indicators.

High-weight keywords within each risk theme were first examined to identify the underlying risk factors. These factors were then grouped according to their risk attributes and mechanisms of action before being further refined with reference to relevant industry standards and engineering practice. This process resulted in a structured risk indicator system capable of representing the principal characteristics of LNG tank truck transportation accidents, while transforming unstructured information in the accident corpus into variables suitable for quantitative analysis (Table 3).

**Table 3:** Mapping of LDA topics, risk dimensions, and representative risk factors.

LDA topic	Risk dimension	Representative risk factors	Description
Topic 1: Accident and Response Process Theme	Accident and emergency response	Accident type, leakage volume, fire and explosion, emergency response time, implementation rate of emergency response measures	Reflects accident evolution and the timeliness and effectiveness of post-event response actions.
Topic 2: Human and Safety-Management Theme	Human factors and safety management	Driver violations, operational errors, safety training coverage, accountability implementation	Reveals the influence of driver behavior and safety-management practices on accident occurrence.
Topic 3: External Environment Theme	Environment and road conditions	Road condition rating, meteorological risk index, traffic volume, frequency of natural hazards	Highlights the influence of environmental and road conditions on transportation safety and emergency response.
Topic 4: Equipment and Facility Theme	Vehicles and infrastructure	Valve reliability, tank structural integrity, maintenance interval, facility maintenance level	Indicates the influence of equipment condition and infrastructure reliability on leakage and accident development.
Topic 5: Institutional and Regulatory Theme	Regulatory and institutional management	Enterprise qualification level, regulatory inspection frequency, regulatory compliance, enforcement intensity	Demonstrates the role of regulatory compliance and institutional oversight in accident prevention and risk control.

Unlike conventional approaches that rely primarily on expert judgment, the proposed framework derives candidate risk indicators from accident-corpus evidence and subsequently refines them through qualitative interpretation and conceptual cross-checking against established hazardous-material transportation and process-safety concepts. This evidence-based process establishes a structured pathway linking the accident corpus, risk themes, risk factors, and risk indicators, thereby enhancing the traceability and conceptual consistency of risk-indicator extraction (19).

Based on the above construction process, the correspondence between the identified risk themes and the extracted risk indicators was established (Table 4). The five risk themes were translated into corresponding categories of risk indicators, which together constitute the risk indicator system for LNG tank truck transportation accidents.

**Table 4:** Mapping between representative topic keywords and risk indicators.

Topic	Representative topic keywords	Primary risk dimension	Representative risk factors (indicators)
Topic 1: Accident and Response Process Theme	Natural gas; LNG tank truck; accident; leakage; firefighting; rear-end collision; fire; rescue	Accident and emergency response	Accident type; accident severity; natural gas leakage volume; emergency response time; implementation rate of emergency response measures
Topic 2: Human and Safety-Management Theme	Accident; expressway; leakage; LNG tank truck; responsibility; unsafe operation; fatigue driving; safety training; accountability	Human factors and safety management	Driver violations; operational errors; safety training coverage; implementation of safety management systems; accountability implementation
Topic 3: External Environment Theme	Safety; vehicle; leakage; rescue; transportation; road conditions; weather conditions; traffic congestion	Environment and road conditions	Road technical grade; traffic flow density; meteorological conditions; frequency of natural hazards
Topic 4: Equipment and Facility Theme	Site; LNG tank truck; transportation; leakage; tank; valve; inspection; maintenance	Vehicles and infrastructure	Vehicle technical condition; tank structural integrity; integrity of safety accessories; equipment inspection and maintenance interval; equipment abnormality records; facility safety assurance
Topic 5: Institutional and Regulatory Theme	Rescue; safety; company; Safety Supervision Bureau; supervision; law enforcement; regulatory implementation	Regulatory and institutional management	Enterprise qualification level; regulatory inspection frequency; implementation of safety regulations; risk assessment systems; emergency response plan coverage

Accident and Response Process Theme describe accident evolution and emergency response capability. Representative indicators include accident type, accident severity, natural gas leakage volume, emergency response time, and on-site emergency response implementation rate, which characterize accident propagation and the severity of accident consequences.

Human and Safety-Management Theme focuses on driver behavior and organizational safety management. Key indicators include driver violations, operational errors, safety training coverage, implementation of safety management systems, and the degree of responsibility implementation, representing the primary targets for accident prevention.

External Environment Theme describes the operating environment of transportation activities. Indicators such as road technical grade, road traffic conditions, traffic flow density, meteorological conditions, and the frequency of natural hazards reflect external conditions that influence both vehicle operational safety and emergency rescue efficiency. These indicators are derived from the present corpus and are therefore not supported by attaching a consequence-modeling citation to the result itself. Their external relevance is instead discussed comparatively: hazardous-material transportation research has long treated road, traffic, and environmental conditions as important risk dimensions, while database-based Natech research shows that natural hazards can act as triggers or amplifiers of hazardous-material releases (20).

The Equipment and Facility Theme characterizes the reliability of transportation equipment. Representative indicators include vehicle technical condition, structural integrity of LNG tanks, operating status of safety accessories, equipment inspection and maintenance intervals, and equipment abnormality records, all of which are closely associated with natural gas leakage and transportation safety.

The Institutional and Regulatory Theme reflects enterprise safety management and government regulatory effectiveness. Corresponding indicators include enterprise qualification level, frequency of safety inspections, implementation of safety regulations, emergency response plan coverage, and the completeness of risk assessment systems, providing institutional support for accident prevention and risk control.

Taken together, the five categories of risk indicators represent different stages of accident occurrence, accident evolution, and accident prevention and control. Their broad content overlaps with established hazardous-material risk research in identifying human, vehicle/equipment, environmental, accident-consequence, and management-related factors. The main difference is methodological and representational: the present framework begins with recurring semantic patterns in unstructured LNG accident narratives and retains emergency response and institutional/regulatory information as explicit dimensions. These findings complement structured root-cause and risk-assessment approaches rather than replacing them. Similar database-learning studies in emerging-energy systems have also shown the value of retaining technical, human, organizational, inspection, maintenance, and safety-barrier information when translating accident experience into preventive action (21).

### 4.4 Consolidated principles and structure of the proposed risk-Indicator framework

The proposed risk-indicator framework links accident-corpus analysis with subsequent health-hazard interpretation and risk-management analysis. To avoid overlap between separate descriptions of “principles” and “characteristics”, the framework is summarized here using five construction criteria: evidence-based relevance, systematic coverage, hierarchical organization, measurability, and LNG-transport specificity. Candidate indicators were first derived from recurring accident-corpus evidence and then refined with reference to hazardous-material transport concepts, applicable road-transport safety requirements, and engineering interpretation. The framework covers accident initiation, accident evolution, emergency response, human and organizational factors, equipment and facilities, external conditions, and institutional oversight. Indicators are organized hierarchically so that broad risk dimensions can be linked to more specific and, where data are available, measurable operational variables.

Importantly, the framework should not be interpreted as a fully validated quantitative risk-assessment instrument. Some indicators, such as collision type, leakage occurrence, road/weather conditions, and reported response actions, are directly observable in the accident corpus; others, including training coverage, inspection frequency, maintenance interval, response-time performance, and inter-agency coordination capability, are proposed operational indicators that require enterprise, regulatory, or emergency-response data for independent measurement and validation. This distinction is consistent with ISO 31000, which treats risk identification, analysis, evaluation, treatment, monitoring, and communication as connected components of a broader risk-management process.

#### 4.4.1 Framework structure

Based on the identified themes and corresponding risk factors, the proposed framework comprises five primary dimensions: Accident and Response Process, Human and Safety Management, External Environment, Equipment and Facilities, and Institutional and Regulatory factors (Table 5). These dimensions transform unstructured accident narratives into a structured set of candidate indicators while preserving the distinction between directly observed corpus evidence and indicators that require external operational data.

**Table 5:** Proposed risk-indicator framework for LNG tank truck transportation accidents.

Primary risk dimension	Secondary risk category	Tertiary risk indicators
Accident occurrence and emergency response risks	Accident characteristics	Accident type, accident severity level, accident frequency
	Leakage dispersion characteristics	LNG leakage volume, leakage duration, affected area
	Emergency response capability	Emergency response time, firefighting arrival time, on-site safety perimeter
	Emergency response effectiveness	Emergency response plan implementation rate, accident control time, occurrence of secondary accidents
Human operation and safety management risks	Driving behavior	Speeding, fatigued driving, illegal lane changing, illegal parking
	Operational compliance	Compliance with loading and unloading procedures, implementation rate of safe operating procedures, number of operational violations
	Safety training	Personnel training coverage, certification rate, frequency of emergency drills
	Enterprise safety management	Accountability implementation, safety inspection frequency, completion rate of hazard rectification
External environmental risks	Road conditions	Road classification, proportion of curved and steep road sections, distribution of bridges and tunnels
	Traffic environment	Traffic density, congestion index, accident-prone level
	Meteorological environment	Rainfall, dense fog, snow and ice conditions, high-temperature weather
	Natural hazards	Landslides, debris flows, rockfall risk, flood risk
Equipment and infrastructure risks	Vehicle technical condition	Vehicle serviceability rate, vehicle service life, equipment failure rate
	Storage and transportation equipment	Tank structural integrity, safety valve condition, pipeline sealing performance
	Maintenance	Maintenance interval, maintenance timeliness, equipment failure records
	Safety devices	Emergency shut-off devices, pressure monitoring devices, operational status of safety alarm systems
Regulatory and institutional governance Risks	Enterprise regulations	Completeness of safety management systems, implementation of safety standards
	Government supervision	Regulatory inspection frequency, enforcement coverage, frequency of special inspection campaigns
	Risk management	Risk assessment system, hazard identification and inspection system, safety evaluation system
	Emergency support	Emergency response plan coverage, emergency resource allocation, inter-agency coordination capability

The framework is therefore intended as an evidence-informed bridge between accident learning and subsequent risk assessment, not as a substitute for FTA, HAZID, bow-tie analysis, or other established causal and scenario-based methods. Its added value lies in systematically screening heterogeneous narrative evidence for recurring factors that can subsequently inform structured risk analysis, targeted data collection, and risk-management priorities (22).

## 5 Health-Hazard interpretation and discussion in relation to risk management

### 5.1 Association analysis between risk identification results and health hazards

The health-hazard analysis separates two evidence layers. First, accident-report evidence includes explicitly documented casualties, exposure circumstances, leakage, fire, explosion, collision, rollover, and emergency-response descriptions. Second, mechanism-based interpretation uses established LNG consequence knowledge to explain how cryogenic contact, thermal radiation, blast effects, or oxygen displacement may lead to frostbite, burns, trauma, or asphyxiation (23). This distinction is important for public-health interpretation: health outcomes are attributed to individual cases only when documented in the source material, whereas other outcomes are presented as technically plausible hazard pathways rather than observed epidemiological effects.

Where the reports documented leakage, fire, explosion, collision, rollover, or rescue exposure, these observations were treated as data-derived accident characteristics. The subsequent pathway from those events to cryogenic injury, thermal burns, blast trauma, or oxygen-deficiency-related asphyxiation was interpreted using established LNG consequence mechanisms. This separation prevents the topic model from being treated as direct epidemiological evidence and is consistent with the broader risk-analysis principle that hazard identification should be distinguished from quantitative estimation of likelihood and consequence.

The identified themes correspond in important respects to factors reported in previous hazardous-material accident studies. Human and safety-management factors in the present corpus are consistent with the prominence of human factors in Chinese hazardous-material road accidents and with recent incident analyses that identify human performance, organizational weaknesses, inspection, maintenance, and operating procedures as recurring contributors (24). Equipment and Facility Theme likewise corresponds to the established importance of containment integrity, valves, seals, inspection, and maintenance in LNG and hydrogen accident learning. The External Environment Theme is consistent with research showing that road, traffic, weather, and natural-hazard conditions can modify hazardous-material accident likelihood or consequences (25).

The principal contribution of the present framework is therefore not the claim that these root causes are entirely new. Rather, it is the integration of recurring accident, response, human, equipment, environmental, and institutional information extracted from heterogeneous Chinese LNG accident narratives into one transparent analytical structure. Compared with structured crash databases, the narrative corpus retains contextual information on emergency actions, responsibility, training, supervision, and post-event response. Compared with FTA/HAZID-type analyses, the framework is data-led at the screening stage and can be used to identify recurrent themes before detailed scenario-specific causal modeling is undertaken (26).

### 5.2 Characteristics of health hazards associated with LNG tank truck transportation accidents

The accident corpus supports recurring events and exposure patterns, while the specific medical consequences discussed below combine documented case information with established LNG hazard mechanisms. Accordingly, this section characterizes potential health-hazard pathways rather than estimating incidence, prevalence, dose–response relationships, or population-level health burden. This bounded interpretation is supported by consequence studies of LNG leakage and dispersion and by clinical evidence from the Zhejiang LNG tanker explosion, which illustrates the severe burn-care demands that can arise in major events.

#### 5.2.1 Multi-factor coupling

LNG tank truck transportation accidents rarely result from a single risk factor. Instead, the corpus shows combinations of human behavior, equipment condition, external environmental factors, and safety-management deficiencies. This multi-factor pattern is consistent with hazardous-material transportation reviews and accident-database studies that treat accident occurrence as the product of interacting driver, vehicle, road/environmental, material, and management factors. Recent hydrogen incident studies similarly report combinations of technical failures, human performance, organizational deficiencies, inspection, maintenance, and safety-barrier weaknesses (27). The present LNG analysis extends this comparison by retaining emergency response as an explicit theme because response activities are highly visible in investigation narratives and directly influence exposure duration.

#### 5.2.2 Chain-like accident evolution

The formation of health hazards can follow a chain-like sequence in which collision, rollover, or equipment failure leads to loss of containment, vapor dispersion, ignition, fire, or explosion. This interpretation is consistent with LNG consequence-modeling research on dispersion, thermal effects, and personnel hazard ranges. However, the LDA topics themselves should not be read as causal chains: they identify recurring semantic co-occurrence, while the causal ordering of events requires manual interpretation and, where appropriate, dedicated methods such as event-tree, fault-tree, bow-tie, or Bayesian analysis.

#### 5.2.3 Compound hazardous exposure

The physicochemical properties of LNG create multiple potential exposure pathways during transportation accidents. Cryogenic contact can cause cold injury; ignition can produce thermal radiation and burns; explosions can cause blast-related trauma; and gas accumulation may displace oxygen in confined or poorly ventilated environments. These mechanisms explain why emergency isolation, detection, firefighting, evacuation, and medical preparedness should be considered together rather than as independent response tasks. Clinical experience from a major LNG tanker explosion further demonstrates the need for coordinated multidisciplinary treatment capacity when severe burn casualties occur (28).

#### 5.2.4 Impacts on public health

The consequences of LNG tank truck accidents may extend beyond drivers and escorts to emergency responders, other road users, and nearby residents. This does not mean that the present study estimates population-level public-health burden; rather, it identifies plausible exposure groups and health-hazard pathways. The distinction is important because hazardous-material releases can create off-site consequences, while the magnitude of exposure depends on release conditions, dispersion, location, population presence, and emergency control. Accordingly, the public-health implication of the present findings lies mainly in preparedness for potentially exposed populations and in reducing the duration and severity of hazardous exposure.

### 5.3 Implications for risk management and governance

Risk-management implications should be interpreted within established frameworks rather than as stand-alone recommendations. ISO 31000 describes risk management as an integrated process involving identification, analysis, evaluation, treatment, monitoring, review, recording, reporting, and communication (29). In parallel, UNDRR and the Society for Risk Analysis distinguish operational risk-management activities from the broader governance arrangements through which responsibilities, information, oversight, and decisions are coordinated (30). On this basis, the recommendations below are organized around source prevention, transport-process control, emergency preparedness/response, and cross-organizational coordination.

First, source prevention should prioritize the recurrent human and equipment factors identified in the corpus. Driver qualification, fatigue management, operating-procedure compliance, safety training, inspection, preventive maintenance, and integrity management are consistent with the main causal categories reported in hazardous-material road-accident research and with database studies of emerging-energy systems, where human performance, maintenance, inspection, component integrity, and organizational measures repeatedly appear as preventive priorities. The present framework adds an LNG road-transport perspective by linking these controls to narrative evidence on driving behavior, containment equipment, and enterprise safety management.

Second, transport-process control should be dynamic because road, traffic, meteorological, and natural-hazard conditions can change during a trip. Route monitoring, weather and hazard warnings, leakage detection, and timely isolation of affected areas are therefore consistent with both hazardous-material transportation risk assessment and Natech research showing that natural hazards can trigger or amplify hazardous-material releases. For the present study, these controls are not inferred from topic weights alone; rather, the topic model identifies recurring environmental and response information that can be combined with operational monitoring data in future quantitative assessment.

Third, emergency response should connect safety management with health protection. LNG consequence studies indicate that dispersion, fire, explosion, and confined-space accumulation can create different exposure zones and injury mechanisms, while clinical evidence from a major LNG tanker explosion highlights the importance of multidisciplinary medical response for severe burns. Consequently, emergency plans should clarify detection, isolation, evacuation, firefighting, responder protection, casualty triage, and medical referral. Where these actions require coordination among transport enterprises, fire and rescue services, transport authorities, regulators, and health services, the issue moves from operational risk management toward risk governance in the sense defined by UNDRR and SRA (31).

Overall, comparison with previous studies shows substantial commonality in the underlying risk factors but a different analytical emphasis. Established hazardous-material and emerging-energy accident studies identify many of the same technical, human, environmental, and organizational contributors. The contribution of the present approach is to use a reproducible TF-IDF-LDA workflow, followed by manual semantic validation, to recover these recurring factors together with emergency-response and institutional information from heterogeneous LNG accident narratives. The proposed indicator framework should therefore be viewed as an evidence-informed screening and structuring tool that complements, rather than replaces, established causal-analysis and risk-management methods. Its indicators require further validation with larger accident databases and enterprise-level operational data before use as a quantitative assessment instrument.

## 6 Conclusion

Using 52 LNG tank truck transportation accident cases reported in China during 2015–2024, this study evaluated a TF–IDF–LDA workflow for extracting recurring themes from accident narratives and supporting structured interpretation of accident-related health hazards. The analysis indicates that reproducible text screening can complement manual review by identifying recurring event, management, environmental, equipment, and institutional patterns, while expert validation remains necessary for distinguishing co-occurrence from causal or conceptual relationships. The public-health relevance of the study lies in the potential exposure of workers, responders, road users, and nearby communities to cryogenic, thermal, blast, and oxygen-deficiency hazards. The low-carbon energy transition provides contextual motivation for the continued relevance of LNG logistics but is not an empirical variable tested in this study.

(1) Accident text mining effectively revealed the risk structure of LNG tank truck transportation accidents. Based on TF–IDF and the LDA topic model, five major risk themes were identified: Accident and Response Process Theme, Human and Safety-Management Theme, External Environment Theme, Equipment and Facility Theme, and Institutional and Regulatory Theme. The identified risk themes span transportation organization, equipment operation, the external environment, and safety management, collectively demonstrating the systematic nature and multi-factor coupling of LNG tank truck transportation accidents. The Human and Safety-Management Theme and the Accident and Response Process Theme were the two most prominent themes in the accident corpus, together accounting for 49.6% of the overall topic weight. In contrast, Equipment and Facility Theme, External Environment Theme, and Institutional and Regulatory Theme continuously influence accident severity by affecting hazardous substance release, accident propagation, and emergency response capability.
(2) The health hazards associated with LNG tank truck transportation accidents exhibit pronounced characteristics of dynamic evolution and public health relevance. Rather than resulting directly from individual risk factors, health hazards develop through successive stages of accident occurrence, hazardous substance release, and hazardous exposure, following the pathway of “risk factors–accident events–hazardous exposure–health outcomes.” The identified risk themes contribute to different stages of accident evolution, collectively shaping the severity of health outcomes through multi-factor coupling, chain-like accident evolution, and compound hazardous exposure. These findings demonstrate that LNG tank truck transportation accidents should be addressed not only as a hazardous goods transportation safety issue but also from the perspectives of occupational health and public health, highlighting the need for full-process risk prevention and control.
(3) The findings indicate that establishing a full-process health risk governance system is essential for improving the safety of LNG tank truck transportation. Effective risk governance should extend beyond post-accident emergency response by integrating accident prevention, transportation process control, emergency response, and health protection into a unified governance framework. Such an approach strengthens source-oriented risk prevention, dynamic transportation risk management, coordinated emergency response, and health protection, thereby promoting the transition from conventional accident management to full-process health risk governance.

Using accident investigation reports as the primary data source, this study established an analytical framework linking “accident corpus–risk themes–health hazards”, extending accident text mining from risk identification to health hazard analysis and risk governance. By integrating accident evolution with a public health perspective, the proposed framework explains how accident risks are transformed into health hazards, providing a new analytical perspective for research on LNG tank truck transportation accidents. Beyond its theoretical contribution, the findings offer practical guidance for transportation enterprises in risk identification, safety management, and emergency preparedness, while supporting government authorities in improving LNG road transportation safety supervision, optimizing hazardous transportation risk prevention and control systems, and strengthening public health risk governance. Consequently, the proposed framework has practical value for enhancing the safety of LNG road transportation under the low-carbon energy transition.

Several limitations remain. The 52-case sample is not statistically representative of all LNG road-transport accidents, and reporting completeness varies across sources. The 1,393 analytical text units are nested within 52 cases and should not be interpreted as independent accidents. LDA identifies patterns of word co-occurrence rather than causal mechanisms, so manual semantic validation is necessary. Some proposed indicators require enterprise, inspection, or emergency-response data that were not consistently available in the accident reports. In addition, the study does not estimate population-level health outcomes, incidence, disease burden, health-economic effects, or the causal health effects of the low-carbon transition. Future research should combine larger accident databases with enterprise operations, road, meteorological, IoT, emergency-response, and health-outcome data to examine exposure and health consequences more directly.

## Data Availability

The original contributions presented in the study are included in the article and its Supplementary material. Further inquiries can be directed to the corresponding author.
